# Anti-MOG Positive Bilateral Optic Neuritis and Brainstem Encephalitis Secondary to COVID-19 Infection: A Case Report

**DOI:** 10.3390/neurolint14040078

**Published:** 2022-11-30

**Authors:** Zisis Tsouris, Antonios Provatas, Christos Bakirtzis, Athina-Maria Aloizou, Vasileios Siokas, Vana Tsimourtou, Nikolaos Grigoriadis, Georgios M. Hadjigeorgiou, Efthimios Dardiotis

**Affiliations:** 1 Department of Neurology, University Hospital of Larissa, Faculty of Medicine, School of Health Sciences, University of Thessaly, 41110 Larissa, Greece; 2 Multiple Sclerosis Center, B’ Department of Neurology, AHEPA University Hospital, Aristotle University of Thessaloniki, 54636 Thessaloniki, Greece; 3 Department of Neurology, Medical School, University of Cyprus, 1678 Nicosia, Cyprus

**Keywords:** COVID-19, case report, Anti-MOG, optic neuritis, brainstem encephalitis

## Abstract

(1) Introduction: There have been numerous reports on the neuroinvasive competence of SARS-CoV-2. Here, we present a case with anti-MOG positive bilateral optic neuritis and brainstem encephalitis secondary to COVID-19 infection. Additionally, we present a review of the current literature regarding the manifestation of anti-MOG positive optic neuritis as well as anti-MOG positive encephalitis after COVID-19 infection. (2) Case Report: A 59-year-old female patient, with a recent history of COVID-19 infection, presented a progressive reduction of visual acuity and bilateral retrobulbar pain for the last 20 days. An ophthalmological examination revealed a decreased visual acuity (counting fingers) and a bilateral papilledema. An MRI scan of the brain revealed a mild thickening of the bilateral optic nerves and high-intensity lesions in the medial and right lateral pons. A high titer of IgG and IgM antibodies against SARS-CoV-2 in serum and antibodies against myelin oligodendrocyte glycoprotein (anti-MOG) in serum and CSF were revealed. The diagnosis of anti-MOG brainstem encephalitis and optic neuritis was set. (3) Conclusions: The history of COVID-19 infection should raise awareness about these autoimmune and infection-triggered diseases, such as anti-MOG antibody disease.

## 1. Introduction

Despite the fact that severe acute respiratory syndrome coronavirus 2 (SARS-CoV-2) mainly affects the respiratory system and produces corresponding respiratory tract symptoms, since the outbreak of the pandemic in December 2019, there have been numerous reports of the neuroinvasive potential of SARS-CoV-2. The disease mechanisms reported so far include the direct infection, para- and post-infectious, as well as vascular, mechanisms [1]. Manifestations such as encephalitis, acute demyelinating encephalomyelitis (ADEM), myelitis, immune-mediated central nervous system (CNS) and peripheral nervous system (PNS) demyelination, and cerebrovascular disease have been reported to date [2,3,4]. However, several other neurological symptoms during the course of the disease have also been reported. Concerning optic neuritis, several cases have been published; however, only six patients were positive for antibodies against myelin oligodendrocyte glycoprotein (anti-MOG). Of these, one patient had a presumed COVID-19 infection, while the rest had a confirmed one [5,6,7,8,9,10]. Moreover, concerning encephalitis, only a few cases with the presence of anti-MOG in serum after COVID-19 infection have been reported [3,4].

Here, we report a case of a female patient with bilateral optic neuritis and brainstem encephalitis secondary to COVID-19 infection. Additionally, we present a review of the current literature regarding the manifestation of anti-MOG positive optic neuritis as well as anti-MOG positive encephalitis after COVID-19 infection.

## 2. Case Presentation

A 59-year-old female patient with a history of hypertension and anxiety disorder, and an unremarkable family history, was referred to the Neurological Emergency Department of the University Hospital of Larissa, a tertiary hospital of central Greece, due to a progressive reduction of visual acuity and bilateral retrobulbar pain for the last 20 days. Forty days prior to the current episode, the patient reported having experienced fever and a cough, and tested positive via a polymerase chain reaction (PCR) test for severe acute respiratory syndrome coronavirus 2 (SARS-CoV-2), from a nasopharyngeal swab. The latter symptoms, attributed to the viral infection, had completely resolved without the need for hospitalization. Informed consent was obtained. Our study follows the principles of the Declaration of Helsinki.

**Figure 1:**
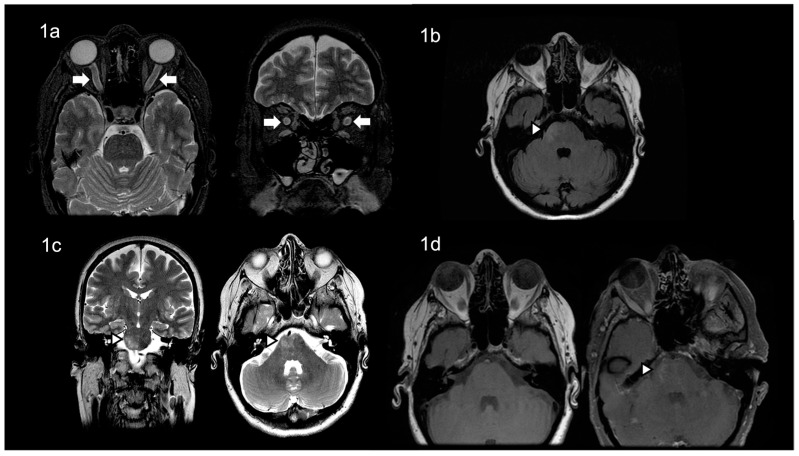
The patient’s brain MRI revealed, in T2 images, (**a**) mild thickening of the bilateral optic nerves, and (**b**,**c**) high-intensity lesions in the medial and right lateral pons, in T2/FLAIR images, (**d**) with contrast enhancement of the above lesions.

The ophthalmological examination revealed a decreased visual acuity (counting fingers) and a bilateral papilledema, while the rest of the neurological examination was unremarkable. A 3 Tesla MRI scan of the brain revealed a mild thickening of the bilateral optic nerves (Figure 1a) and high-intensity lesions in the medial and right lateral pons (Figure 1b–d).

Routine blood tests presented no remarkable abnormalities, while serological studies with autoimmune markers, including antinuclear and anti-dsDNA antibodies, and the rheumatoid factor were not suggestive of other autoimmune diseases. A high titer of IgG and IgM antibodies against SARS-CoV-2 was detected in serum. An initial cerebrospinal fluid (CSF) analysis showed 7 cells/mm3 as well as normal protein (34.5 mg/dL) and glucose (50.9 mg/dL) levels. In addition, a CSF analysis with PCR for several viral and bacterial infections (Mumps virus, Measles virus, Human enterovirus, Parechovirus, Herpes simplex virus 1, Herpes simplex virus 2, Varicella zoster virus, Epstein–Barr virus, Cytomegalovirus, Human herpes virus 6, 7 and 8, *Listeria monocytogenes*, *Heamophilus influenzae*, *Staphylococcus aureus*, *Streptococcus pneumoniae*, *Streptococcus agalactiae*, *Neisseria meningitis*, *Borrelia burgdorferi*, *Escherichia coli* K1, *Cryptococcus neoformans*, *Cryptococcus gatii*, West Nile virus, and SARS-CoV-2) was negative. The oligoclonal band status in CSF was negative. Furthermore, antibodies against myelin oligodendrocyte glycoprotein (anti-MOG) were revealed in serum (1/32) and CSF. According to the above, the diagnosis of anti-MOG brainstem encephalitis and optic neuritis was set.

Consequently, the patient was treated with 1000 mg of intravenous (IV) methylprednisolone for 5 days and oral phenytoin 4 mg/kg/day for 7 days as a retinoprotective agent [11]. Due to no significant clinical improvement, the patient additionally received intravenous immunoglobulin (IVIg) 2 g/kg, besides the oral prednisolone treatment. Following the treatment, the neurological examination showed no deficits, and the ophthalmological examination showed improvement in the visual acuity (6/10 in the right eye, 7/10 in the left eye) and a remission of the bilateral papilledema. The patient was therefore discharged with a prescription for oral prednisolone (30 mg/day).

## 3. Discussion

A comprehensive literature review of reported optic neuritis and COVID-19 infection cases was performed, including as many patients as possible. We used the PubMed database, with the following search terms: ‘’optic neuritis’’, ‘’MOG’’, ‘’encephalitis’’ and “COVID-19”. Additional articles were identified by hand-searching references of the included literature (Table 1).

MOG antibody disease is mediated via antibodies against MOG, expressed on oligodendrocytes. MOG antibody disease is responsible for clinical manifestations like optic neuritis, transverse myelitis, encephalitis, and ADEM. Anti-MOG antibodies may be present in the bloodstream without causing symptoms until they enter the CNS. Along with lung cells, which are the main target of the SARS-CoV-2 virus, endothelial cells forming the blood–brain barrier (BBB) also express the angiotensin-converting enzyme 2 receptors (ACE2). By infecting these cells through ACE2 receptors, SARS-CoV-2 causes inflammation of the endothelium and the subsequent breakdown of the BBB. As a result, both anti-MOG and leukocytes can cross the BBB and trigger the onset of the disease [12,13].

Our case report adds to the existing bibliography on the neurological sequelae of COVID-19 [4]. With the ever-rising number of infected patients, it is reasonable to expect that these entities will also be encountered more frequently, and so clinicians must act promptly in diagnosing and treating patients. Therefore, a history of COVID-19 infection should raise awareness about these autoimmune and infection-triggered diseases, such as anti-MOG antibody disease.

## 4. Conclusions

Our case expands the spectrum of SARS-CoV-2 neurological disorders, since we here present a MOG-associated encephalitis and anti-MOG optic neuritis secondary to COVID-19 infection. While the incidence of these neuroimmune manifestations is low, early identification and initiation of corticosteroid therapy is essential to avoid disability. Despite the attempts of the international medical community to record all clinical manifestations of SARS-CoV-2, it seems that the emergence of new neuroimmunological manifestations after COVID-19 infection should raise awareness about MOG-related disease.

## Figures and Tables

**Table 1:** Characteristics of patients with anti-MOG positive optic neuritis and anti-MOG encephalitis secondary to COVID-19 infection.

Author (Year)	Age (Years)/Ethnicity/Sex (Male/Female)	Method of COVID-19 Diagnosis	COVID-19 Symptoms	Hospitalization (Yes/No)	Time of Onset of Neurological Manifestations from COVID-19 Diagnosis	Neurological Manifestations	MOG-Antibody Method of Detection	Treatment	Outcome
de Ruijter et al. (2020)	15/Caucasian/Male	not confirmed	fever, nausea and cough	NM	Few weeks	Subacute vision loss with photopsias and frontal continuous headache	NM	IVMP 1 g/day for three days	Symptoms resolved
Zhou et al. (2020)	26/Hispanic/Male	nasal and oropharyngeal swabs (RT-PCR)	dry cough	Yes	Few days	Bilateral, subacute, sequential vision loss first affecting the left eye, then the right eye 3 days later	MOG-IgG cell-based assays	IVMP 1 g/day for five days	Visual acuity improved rapidly
Sawalha et al. (2020)	44/Hispanic/Male	nasopharyngeal swabs (RNA PCR) and serum (IgG abs)	shortness of breath and cough	No	Two weeks	Right eye pain that had progressed to his left eye along with bilateral blurring of vision leading to a complete vision loss	NM	IVMP 1 g/day for five days	Complete restoration of vision in the left eye with remarkable but not complete vision recovery in the right eye
Zoric et al. (2021)	63/NM/Male	serology was positive for IgM and IgG antibodies against the virus	fatigue, shortness of breath, dry cough and fever	Yes	Four weeks	Right eye blurred vision	Indirect immunofluorescence (MOG antibodies)	IVMP 1 g/day for five days with prednisone tapering therapy for two weeks	Visual acuity was improved, and disk edema was resolved entirely
Khan et al. (2021)	11/NM/Male	nasopharyngeal swab was positive by CBNAAT	redness and ophthalmodynia in both eyes four days after a brief febrile illness	Yes	Two weeks	Loss of vision in the right eye	NM	Pulse methylprednisolone with oral steroids continued and tapered over 12 weeks	Visual acuity was improved
Kogure et al. (2021)	47/Japanese/Male	nasal and oropharyngeal swabs (PCR)	asymptomatic	Yes	N/A	Left eye pain and an upper-visual-field defect	MOG-immunoglobulin G (MOG-IgG) testing in blood	IVMP 1 g/day for a total of 3 days, followed by an oral prednisolone tape	Visual acuity subsequently improved
Peters et al. (2021)	23/NM/Male	nasopharyngeal PCR testing	asymptomatic	Yes	N/A	Progressive headache associated with dysesthesias fatigue, inattention, cognitive slowing, fevers, generalized seizures	MOG-IgG via FACS	IVMP 1 g/day for five days	Cognitive improvement
Durovic et al. (2021)	22/NM/Male	PCR testing	severe headache, fever, neck stiffness, general weakness, and a loss of smell and taste		Ten days	Headache, neck rigidity	Serum MOG-IgG (live-cell assay) *	IVMP 1 g/day for five days	Symptoms resolved

* Serum studies also revealed a low metabotropic glutamate receptor 1 (mGluR1) antibody titer (fixed cell assay, 1:40); CBNAAT, Cartridge Based Nucleic Acid Amplification Test; PCR, polymerase chain reaction; MOG, myelin oligodendrocyte glycoprotein; IVMP, intravenous methylprednisolone; NM, not mentioned; N/A, not applicable; FACS, Fluorescence-Activated Cell Sorting.

## Data Availability

Not applicable.
